# White Blood Cell/Mean Platelet Volume Ratio as a Predictor of Long-Term Outcomes but Not Coronary Artery Disease Severity in Non-ST Elevation Myocardial Infarction Patients

**DOI:** 10.7759/cureus.51423

**Published:** 2023-12-31

**Authors:** Gizem Demir, Gürkan Karaca, Ahmet Ekmekci, Seyedehtina Safaei, Ali Kimiaei, Ayşe Emre

**Affiliations:** 1 Department of Cardiology, Dr. Siyami Ersek Thoracic and Cardiovascular Surgery Educational Research Hospital, Istanbul, TUR; 2 Department of Cardiology, Bahçeşehir University, Istanbul, TUR; 3 Department of Cardiology, VM Medical Park Maltepe Hospital, Istanbul, TUR; 4 Department of Cardiology, Medical Park Pendik Hospital, Istanbul, TUR

**Keywords:** non-st-elevation myocardial infarction (nstemi), long-term outcomes, syntax score, wmr, wbc/mpv ratio

## Abstract

Background

White blood cell count (WBC) and mean platelet volume (MPV) have been shown to be hematologic parameters of prognostic significance in acute coronary syndrome. We sought to determine the relationship between the WBC/MPV ratio (WMR), coronary artery disease (CAD) complexity, and long-term clinical outcomes in patients with non-ST elevation myocardial infarction (NSTEMI).

Hypothesis

WMR has a relationship with the complexity of CAD and long-term clinical outcomes in NSTEMI patients.

Methods

A total of 289 NSTEMI patients who underwent coronary angiography were divided into two groups according to the median WMR (>970 or ≤970). CAD complexity was assessed with the SYNTAX score.

Results

The WMR was not associated with the synergy between percutaneous coronary intervention (PCI) with Taxus and cardiac surgery (SYNTAX) score on multivariate linear regression analysis (ß=0.08, 95% CI = -0.76-2.21, p = 0.14). However, it was of prognostic significance on Kaplan-Meier survival analysis in overall patients (log-rank p = 0.03) and in patients with a SYNTAX Score <22 (log-rank p = 0.01). Follow-up data showed that major adverse cardiac events (MACE) (p = 0.02), death (p < 0.001), non-fatal MI (p = 0.03), ischemia-driven revascularization (p = 0.03), and heart failure (p = 0.04) were more frequent in the high WMR group. After adjustment for age, sex, eGFR, troponin levels, and the Global Registry of Acute Coronary Events (GRACE) score in Cox regression models, the association of high WMR with the cumulative incidence of MACE was preserved (overall patients (HR=1.85, 95% CI 1.1-3.12, p=0.02) and patients with a SYNTAX score <22 (HR=2.06, 95% CI 1.15-3.67, p=0.01).

Conclusion

The WMR was not related to CAD complexity, but it was associated with long-term clinical outcomes in patients with NSTEMI who underwent coronary angiography.

## Introduction

Non-ST-segment elevation myocardial infarction (NSTEMI) is the most prevalent type of acute coronary syndrome (ACS) and accounts for the majority of patients who undergo percutaneous coronary intervention for this condition.

Patients with NSTEMI have a high risk of long-term adverse outcomes comparable to those of patients with ST-segment elevation myocardial infarction (STEMI) [1]. Inflammation and platelet activation are key pathophysiological mechanisms in ACS, which can be reflected by hematological parameters such as white blood cell (WBC) count and mean platelet volume (MPV). Previous studies have indicated that elevated WBC and MPV are associated with increased mortality in patients with ACS [2-5].

The synergy between PCI with Taxus and cardiac surgery (SYNTAX) score is a validated tool that quantifies the extent and complexity of coronary artery disease (CAD) based on angiographic findings. It has prognostic implications and provides therapeutic guidance for revascularization strategies in patients with ACS [6-8]. However, the relationship between the WBC/MPV ratio (WMR), a novel marker of inflammation and platelet activation, and the SYNTAX score, as well as the impact of WMR on the severity of CAD and long-term outcomes in NSTEMI patients, has not been previously explored.

## Materials and methods

Study population

We conducted a retrospective cohort study of 289 patients with NSTEMI who underwent coronary angiography at Dr. Siyami Ersek Thoracic and Cardiovascular Surgery Educational Research Hospital between February 2017 and December 2018. The study was approved by the hospital's Specialty and Education Committee and the Ministry of Health, Health Sciences University, in accordance with the Helsinki Declaration. We defined NSTEMI according to the European Society of Cardiology (ESC) Guidelines for the Management of ACS in patients presenting without persistent ST-segment elevation [9]. We excluded patients with a history of coronary revascularization (bypass surgery or percutaneous intervention), severe liver disease, malignancy, blood transfusion, hematological disease, acute or chronic infection, autoimmune disease, or immunosuppressive therapy.

Data collection

We collected demographic, clinical, laboratory, and treatment data from the hospital’s electronic database. The WMR was calculated by dividing the WBC count by the MPV in units of 10^3^/µL. Patients were stratified into two groups based on the median WMR (>970 or ≤970). Complete blood counts were measured using an automated hematology analyzer (MINDRAY BC-6800, China), and biochemistry parameters were assessed using ARCHITECT PLUS CI-4100 (USA) from venous blood samples collected within 60 minutes of admission. The left ventricular ejection fraction (LVEF) was assessed by echocardiography at admission. The Global Registry of Acute Coronary Events (GRACE) risk score was calculated to categorize patients into three risk groups (<109, 109-140, >140) based on factors such as age, heart rate, systolic blood pressure, serum creatinine level, Killip class, cardiac arrest, ST-segment deviation, and elevated cardiac markers [10]. The prevalence and severity of CAD were evaluated using the SYNTAX score, calculated by two experienced cardiologists [6].

Follow-up

Patients were followed for a median of 311 days after discharge through direct hospital admissions or telephone conversations with the patient or their family. Major adverse cardiac events (MACE), defined as mortality, myocardial infarction (MI), revascularization due to ischemia, and hospitalization for heart failure, were noted. MI was defined according to the universal definition of myocardial infarction [11]. All patients received standard antiplatelet therapy with aspirin and clopidogrel.

Statistical analysis

We performed statistical analysis using IBM SPSS Statistics for Windows, Version 22 (Released 2013; IBM Corp., Armonk, New York). We considered a two-tailed p-value of less than 0.05 as statistically significant. Categorical variables were expressed as frequency (percentage) and continuous variables as mean ± SD after assessing normal distribution using the Shapiro-Wilk test. We compared quantitative data that did not exhibit a normal distribution using the Mann-Whitney U test and categorical variables using the chi-square test or Fisher's exact test. Group means of continuous variables were compared using the independent samples t-test. A general linear model was used to perform a single-variable analysis to identify indicators for the SYNTAX score. Variables with a p-value <0.15 were included in the multivariate regression model analysis, and a p-value <0.05 was considered significant in the multivariate model. The entire patient group and then the patients based on the SYNTAX score (<22 or ≥22) were divided based on the cumulative MACE incidence. Survival analysis was conducted using the Kaplan-Meier method and compared using the log-rank test. Unadjusted and adjusted hazard ratios were calculated using Cox regression models. Variables with a p-value <0.20 in the univariate models were considered as potential confounding factors, and factors with a p-value <0.05 were retained in the final model.

## Results

We included 289 consecutive NSTEMI patients (mean age 56.4±11.8 years, 68.5% male) who underwent coronary angiography in our study. The baseline characteristics, treatment strategies, and clinical outcomes of the patients according to the WMR (<970 or ≥970) are as follows (Table 1).

**Table 1:** Key variables, treatment strategies, and outcomes in patients undergoing treatment. ACEI: angiotensin-converting enzyme inhibitor; ARB: angiotensin receptor blocker; MPV: mean platelet volume; RDW: red blood cell distribution width; WBC/MPV: mean platelet volume to white blood cell ratio; SYNTAX: synergy between PCI with Taxus and cardiac surgery; PCI: percutaneous coronary intervention; MACE: major adverse cardiac events.

Variables	All, n = 289	WBC/MPV > 970, n = 146	WBC/MPV < 970, n = 143	P-value
Age, year (mean ± SD)	56.4 ± 11.8	54.7 ± 12.4	58.3 ± 10.9	0.01
Male, n (%)	198 (68.5)	101 (0.69)	97 (67)	0.80
Hypertension, n (%)	129 (44.6)	61 (41.8)	68 (47.6)	0.32
Past coronary artery disease, n (%)	36 (12.5)	13 (8.9)	23 (16.1)	0.07
Peripheral artery disease, n (%)	2 (0.7)	1 (0.7)	1 (0.7)	1.00
Passed cerebrovascular event, n (%)	3 (1)	1 (0.7)	2 (1.4)	0.98
Diabetes mellitus, n (%)	90 (31)	43 (29.5)	47 (32.9)	0.53
Dyslipidemia, n (%)	139 (48.1)	64 (43.8)	75 (52.4)	0.14
Obesity, n (%)	94 (32.5)	43 (29.5)	51 (35.7)	0.26
Smoking history, n (%)	213 (73.7)	114 (78.1)	99 (69.2)	0.08
Medication history				
Aspirin, n (%)	29 (10)	12 (8.2)	17 (12)	0.29
Beta blocker, n (%)	32 (11.1)	14 (9.6)	18 (12.6)	0.41
Statin, n (%)	19 (6.6)	6 (4.1)	13 (9.1)	0.08
ACEI/ARB, n (%)	66 (22.8)	26 (17.8)	40 (28)	0.04
Angina, n (%)	270 (93.4)	134 (91.8)	136 (95.1)	0.25
Killip classification, n (%)	3 (1)	3 (2)	0 (0)	0.22
Resuscitation, n (%)	1 (0.3)	1 (0.7)	0 (0)	1
Heart rate, bpm (mean ± SD)	81.6 ± 14.6	82.5 ± 15.3	80.7 ± 13.8	0.31
Systolic BP, mmHg (mean ± SD)	135.9 ± 19.2	133.5 ± 19.5	138.3 ± 18.6	0.03
ST segment deviation, n (%)	105 (36.3)	57 (39)	48 (33.6)	0.33
Troponin I, ng/µL	1.04 (7.5)	2.2 (16)	0.5 (2.9)	<0.01
GFR, mL/min/1.73 m² (mean ± SD)	88.5 ± 20	85.7 ± 18.8	91.3 ± 20.8	0.02
Left ventricular ejection fraction (LVEF), (%) (mean ± SD)	53.6 ± 9.1	54.1 ± 9.2	53.2 ± 96	0.46
GRACE score				
<109, n (%)	214 (74)	109 (74.7)	105 (73.4)	0.80
109–140, n (%)	68 (23.5)	31 (21.2)	37 (25.9)	0.70
<140, n (%)	7 (2.4)	6 (4.1)	1 (0.7)	0.12
Total cholesterol, mg/dL (mean ± SD)	193.6 ± 47.8	195.7 ± 51.1	191.4 ± 44.4	0.45
LDL, mg/dL (mean ± SD)	121.7 ± 38.5	123.4 ± 39.7	119.9 ± 37.4	0.46
HDL, mg/dL (mean ± SD)	35.5 ± 9.39	35.1 ± 9.4	35.9 ± 9.3	0.46
Triglyceride, mg/dL (mean ± SD)	154 (98)	152 (94)	157.5 (108)	0.98
Glucose, mg/dL (mean ± SD)	119.5 ± 47.5	119.3 ± 46.5	119.8 ± 48.7	0.93
Serum creatinine, mg/dL (mean ± SD)	0.9 ± 0.5	0.95 ± 0.18	0.89 ± 0.2	0.41
White blood cell count, × 10³/µL (mean ± SD)	9.6 ± 2.9	11.8 ± 2.5	7.4 ± 1.3	<0.01
Neutrophil, × 10³/µL (mean ± SD)	6.3 ± 2.6	8.1 ± 2.4	4.5 ± 1.2	<0.01
Lymphocyte, × 10³/µL (mean ± SD)	2.5 ± 0.9	2.8 ± 1.1	2.2 ± 0.7	<0.01
Hemoglobin, g/L (mean ± SD)	13.9 ± 1.7	14.1 ± 1.8	13.6 ± 1.6	<0.02
Red blood cell count, × 10^6^/µL (mean ± SD)	4.6 ± 0.5	4.8 ± 0.5	4.6 ± 0.5	<0.01
Platelet count, 10^9^/L × 1000/µ/L (mean ± SD)	256.9 ± 86	281.9 ± 100	231.4 ± 59.4	<0.01
MPV, fL (mean ± SD)	9.5 ± 1.1	9.9 ± 0.9	9.3 ± 1.1	<0.01
RDW, % (mean ± SD)	13.7 ± 1.5	13.8 ± 1.7	13.7 ± 1.4	0.53
WBC/MPV, 10³/µL/fL (mean ± SD)	1.04 ± 0.3	1.2 ± 0.3	0.8 ± 0.1	<0.01
Multivessel disease, n (%)	118 (40.8)	56 (38.4)	62 (43.4)	0.38
Mean SYNTAX score, n (%)	9 (15)	9 (15.6)	8 (17)	0.37
SYNTAX score>22	61 (21.1)	32 (21.9)	29 (20.3)	0.73
Revascularization, n (%) (CABG or PCI)	166 (57.4)	86 (58.9)	80 (55.9)	0.61
Only PCI performed, n (%)	122 (42.2)	64 (43.8)	58 (40.6)	0.57
In-hospital medications	288 (99.3)	146 (100)	142 (98.6)	0.15
Aspirin, n (%)	271 (93.4)	135 (92.5)	136 (94.4)	0.49
P2Y2 blocker, n (%)	280 (96.6)	142 (97.3)	138 (95.8)	0.50
Beta blocker, n (%)	277 (95.5)	139 (95.2)	138 (95.8)	0.79
Statin, n (%)	275 (94.8)	136 (93.2)	139 (96.5)	0.19
In-hospital follow-up				
Length of stay, days	5 (3)	4 (3)	5 (3)	0.69
MACE, n (%)	16 (5.5)	11 (7.5)	5 (3.4)	0.25
Mortality, n (%)	7 (2.4)	5 (3.4)	2 (1.4)	0.26
Reinfarction, n (%)	1 (0.3)	0 (0)	1 (0.7)	0.49
Major bleeding, n (%)	7 (2.4)	6 (4.1)	1 (0.7)	0.04
Acute heart failure, n (%)	1 (0.3)	0 (0)	1 (0.7)	0.49
Outpatient follow-up				
Follow-up duration, days (mean ± SD)	283 (171)	311 (175)	265.5 (190.7)	0.15
MACE, n (%)	68 (23.5)	42 (28.7)	26 (18.1)	0.02
Mortality, n (%)	10 (3.5)	9 (6.2)	1 (0.7)	<0.01
Non-fatal myocardial infarction, n (%)	19 (6.6)	10 (6.8)	9 (6.3)	0.03
Ischemia-related revascularization, n (%)	31 (10.7)	16 (11)	15 (10.5)	0.03
Acute heart failure, n (%)	8 (3)	7 (5)	1 (0.8)	0.04

Patients with a high WMR were younger (p = 0.01) and had higher rates of ACEI/ARB use (p = 0.04) than those with a low WMR. They also had lower baseline systolic blood pressure (p = 0.03), lower eGFR values (p = 0.02), and higher troponin I levels (p<0.001). There were no significant differences in cardiovascular risk factors and other baseline findings between the two groups. The laboratory values revealed that the high WMR group had higher levels of WBC (p<0.001), neutrophils (p<0.001), lymphocytes (p<0.001), hemoglobin (p=0.02), red blood cell count (p<0.001), platelet count (p<0.001), and MPV (p<0.001) than the low WMR group. The distribution of the GRACE risk score categories (<109, 109-140, and >140) was similar in both groups. The angiographic findings showed no significant differences in the proportion of patients with multivessel disease and a SYNTAX score >22 between the two groups. The rates of coronary revascularization (bypass surgery or percutaneous intervention) were also comparable in both groups. The in-hospital medication treatment was similar in both groups. The length of hospital stay and the incidence of MACE, death, MI, and heart failure during hospitalization were not different between the two groups. However, major bleeding was more frequent in patients with a high WMR (p = 0.04).

**Figure 1:**
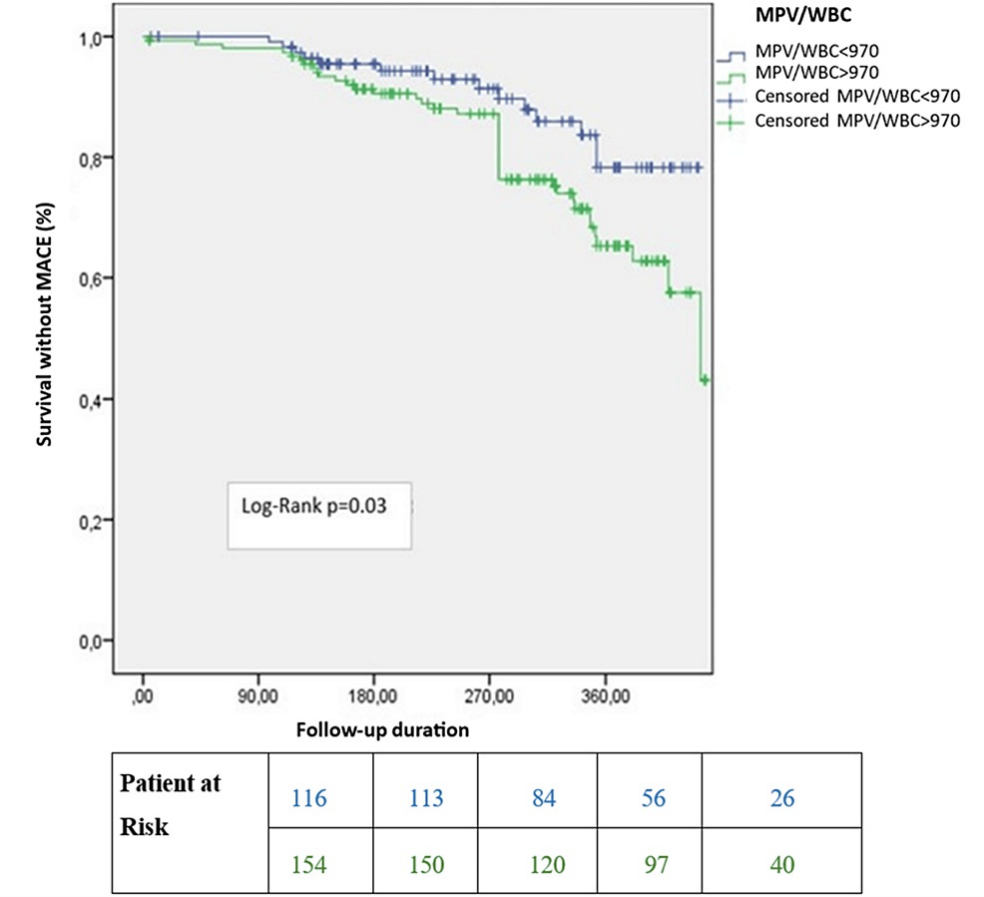
Kaplan-Meier survival curves after discharge in all patients. MPV/WBC: mean platelet volume-to-white blood cell ratio; MACE: major adverse cardiac events.

**Figure 2:**
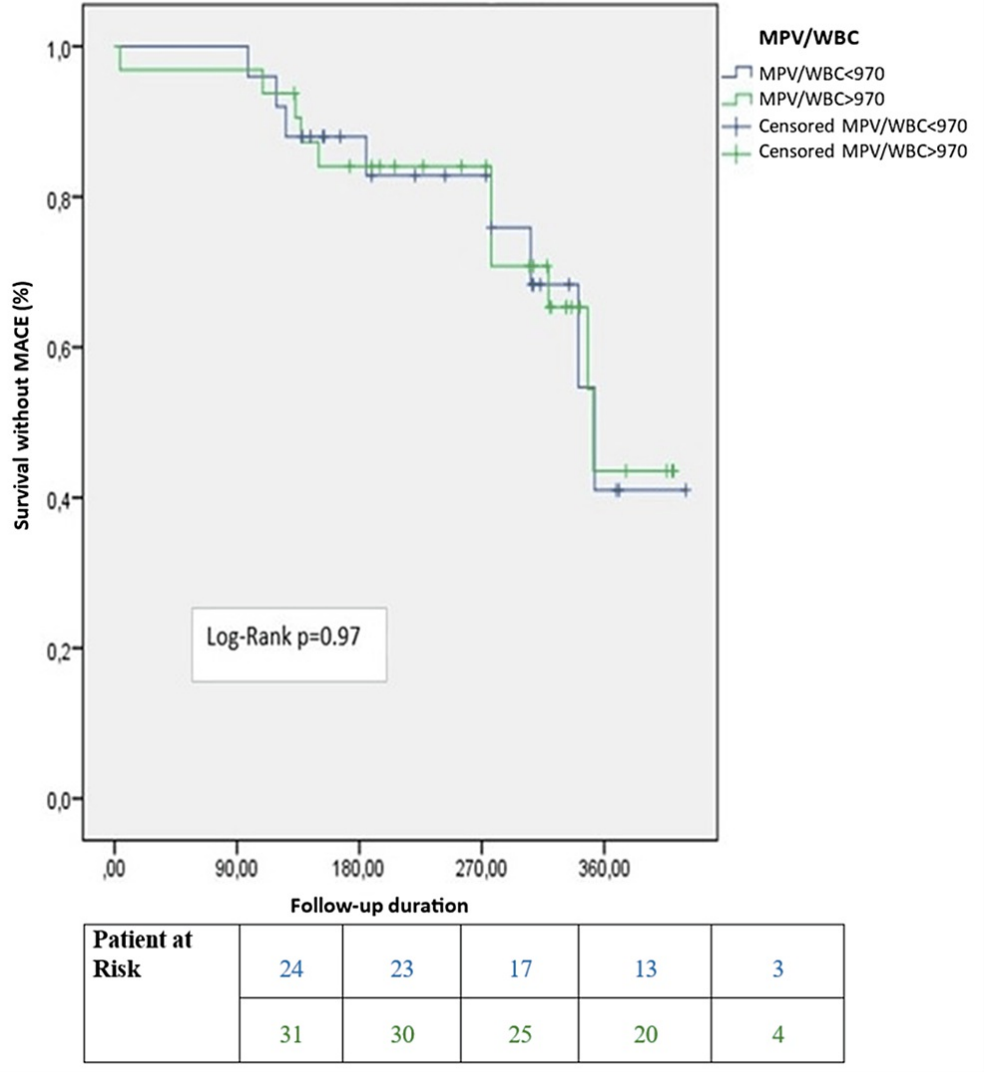
Kaplan-Meier survival curves after discharge in patients with a SYNTAX score greater than 22. MPV/WBC: mean platelet volume-to-white blood cell ratio; SYNTAX: synergy between percutaneous coronary intervention with Taxus and cardiac surgery; MACE: major adverse cardiac events.

**Figure 3:**
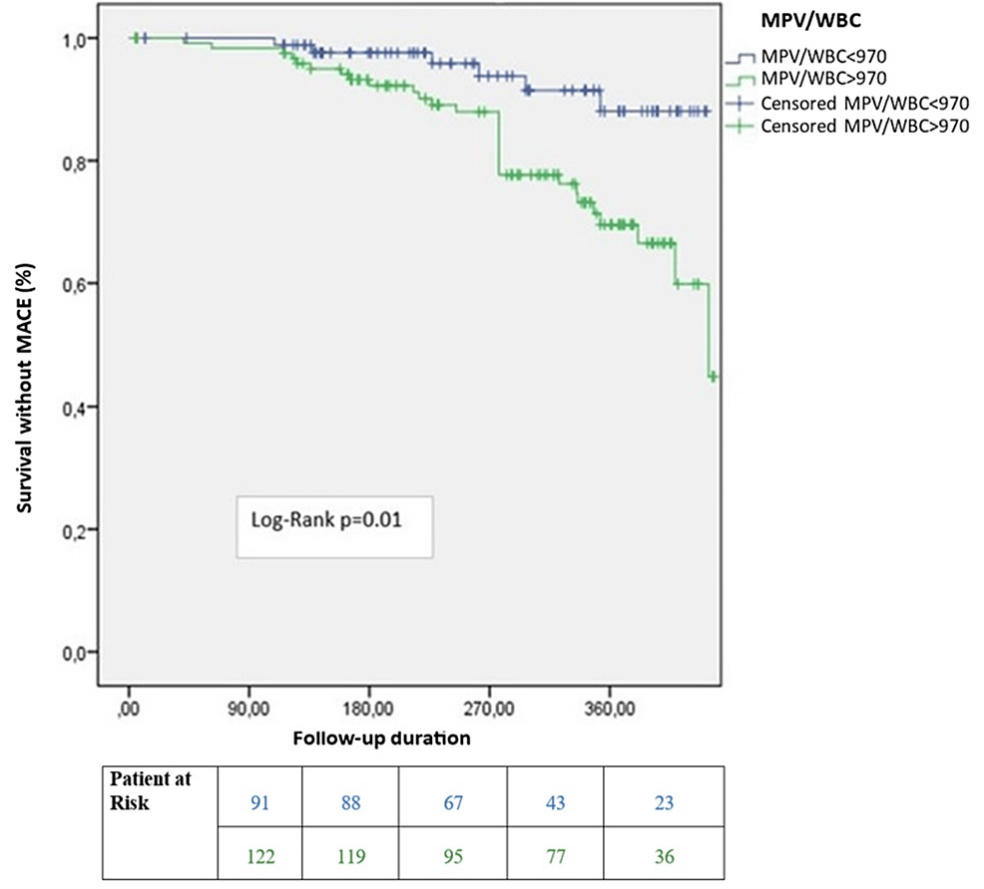
Kaplan-Meier survival curves after discharge in patients with a SYNTAX score less than 22. MPV/WBC: mean platelet volume-to-white blood cell ratio; SYNTAX: synergy between percutaneous coronary intervention with Taxus and cardiac surgery; MACE: major adverse cardiac events.

**Figure 4:**
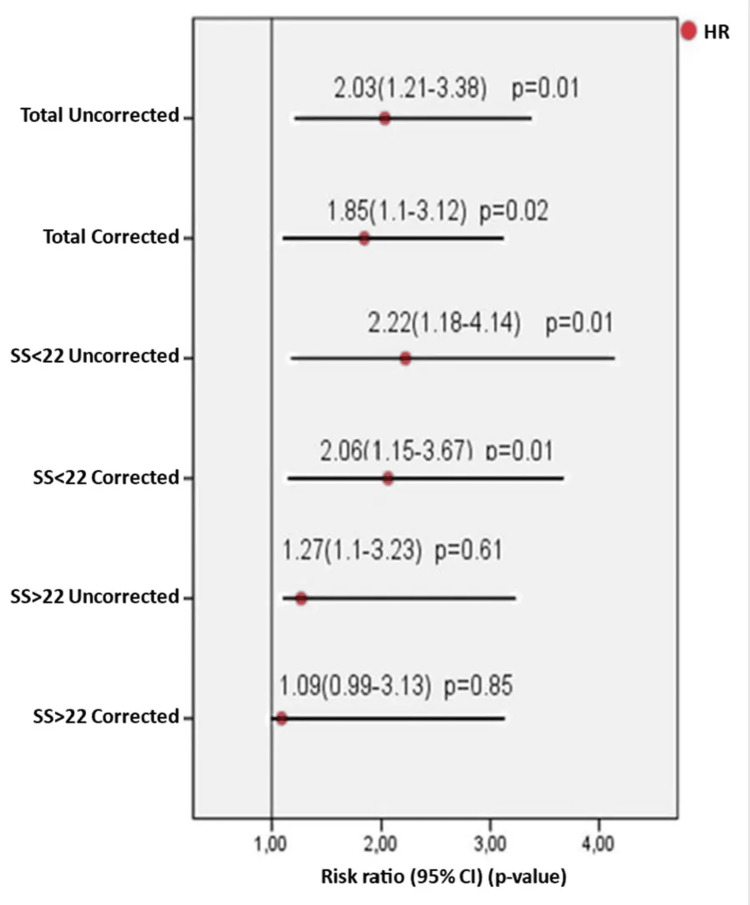
Association of WMR with MACE after adjustment for age, gender, eGFR, troponin, and GRACE score in the Cox regression model. SS: SYNTAX score; WMR: White blood cell count-to-mean platelet volume ratio; MACE: major adverse cardiac events; eGFR: estimated glomerular filtration rate; GRACE: The Global Registry of Acute Coronary Events.

In multivariate linear regression analysis, troponin I levels were not associated with the SYNTAX score (ß = 0.08, 95% CI = -0.76-5.21, p = 0.14). The WMR was also not correlated with the SYNTAX score (ß = 0.12, 95% CI = 0.00-0.09, p = 0.04). However, it was a significant predictor of long-term survival (median 311 days) in all patients (log-rank p = 0.03) and in patients with a SYNTAX score <22 (log-rank p = 0.01) (Figures 1, 2). Notably, it was not a significant predictor in patients with a SYNTAX score >22 (log-rank p=0.97) (Figure 3). In the long-term follow-up after discharge, the high WMR group had higher rates of MACE (p = 0.02), mortality (p < 0.001), MI (p = 0.03), ischemia-related revascularization (p = 0.03), and heart failure (p = 0.04) than the low WMR group. After adjusting for age, sex, eGFR, troponin, and GRACE score in Cox regression models, the high WMR remained an independent predictor of MACE in all patients (HR = 1.85, 95% CI 1.1-3.12, p = 0.02) and in patients with a SYNTAX score <22 (HR = 2.06, 95% CI 1.15-3.67, p = 0.01) (Figure 4).

## Discussion

This study evaluated the prognostic value of WMR in NSTEMI patients who underwent coronary angiography. The main findings of this study are as follows: (1) The WMR was not related to the SYNTAX score, which measures CAD complexity, but it was a significant predictor of long-term mortality and MACE in NSTEMI patients; (2) The high WMR group had higher rates of MACE, death, MI, ischemia-driven revascularization, and heart failure than the low WMR group during the median follow-up of 311 days, and this association remained significant after adjusting for potential confounders; (3) The high WMR group had more major bleeding events than the low WMR group during hospitalization, but there were no differences in other in-hospital outcomes between the two groups.

Hematological markers are easily and cheaply measured by a complete blood count test and have been shown to have prognostic value in ACS patients [11-17]. Previous studies have demonstrated that the NLR, PLR, and WMR are associated with short- and long-term mortality and MACE in ACS patients [12,14,17]. Moreover, MPV has been established as a prognostic marker in NSTE-ACS patients [13,15]. WBC is an indicator of inflammation and hypercoagulability in ACS and plays a role in myocardial damage [2,3,11]. Elevated WBC levels have been associated with increased six-month mortality rates in NSTE-ACS patients [4].

MPV reflects platelet activation and thrombogenic potential [18] and has been linked to long-term adverse events and mortality in NSTEMI patients who underwent percutaneous coronary intervention [5]. In a recent study examining the effects of different hematological parameters on the prediction of 30-day mortality and MACE risk in ACS patients, the WMR was found to be the strongest predictor of both outcomes, compared to WBC, MPV, NLR, PLR, and EDG [19]. The authors suggested that increased platelet activation in the high WMR group led to higher WBC levels and maintained a high WMR.

In our study, we focused on a more homogeneous patient group of NSTEMI patients who underwent coronary angiography. We found that the high WMR group had higher levels of WBC, MPV, neutrophils, lymphocytes, hemoglobin, red blood cell count, and platelet count than the low WMR group. These findings indicate that the high WMR group had more inflammation and platelet activation than the low WMR group. We also found that the high WMR group experienced more major bleeding events than the low WMR group during hospitalization. This may be explained by the higher platelet activation and thrombogenicity in the high WMR group, which may increase the risk of bleeding complications after antiplatelet therapy or invasive procedures. However, we did not find any differences in other in-hospital outcomes between the two groups. This may be due to the short duration of hospitalization and the similar treatment strategies in both groups.

The most important finding of our study was that the WMR was not correlated with the SYNTAX score, a validated tool that quantifies CAD severity based on angiographic findings. This suggests that the WMR does not reflect CAD complexity but rather inflammation and platelet activation. However, we found that the WMR was a significant predictor of long-term survival and MACE in NSTEMI patients. This suggests that the WMR may have prognostic implications for NSTEMI patients, regardless of CAD severity. We also found that this association was independent of age, sex, eGFR, troponin, and GRACE score, which are known risk factors for adverse outcomes in NSTEMI patients. Moreover, we found that the WMR was especially predictive of long-term outcomes in patients with a SYNTAX score <22, indicating low to intermediate CAD complexity. This may imply that the WMR can identify high-risk patients who may benefit from more aggressive treatment or closer follow-up among those with less severe CAD.

Our study has some limitations that should be considered. First, the SYNTAX score only evaluates vessels with a diameter ≥1.5 mm and ≥50% stenosis and may not capture all aspects of CAD severity. Second, we only used baseline complete blood count results and did not assess changes in hematological parameters during follow-up. Third, we did not measure other proinflammatory markers such as P-selectin, C-reactive protein, interleukin 6 and 8, and oxidative stress parameters that may be related to the WMR.

## Conclusions

In conclusion, this study demonstrated that the WMR is a significant predictor of long-term mortality and MACE in NSTEMI patients who underwent coronary angiography, independent of the SYNTAX score, which measures CAD complexity. The high WMR group had higher rates of MACE, death, MI, ischemia-driven revascularization, and heart failure than the low WMR group during the median follow-up of 311 days. Additionally, the high WMR group experienced more major bleeding events than the low WMR group during hospitalization. These findings suggest that the WMR reflects inflammation and platelet activation, key pathophysiological mechanisms in ACS. The WMR may serve as a simple and effective method for identifying high-risk patients who may benefit from more aggressive treatment or closer follow-up, particularly among NSTEMI patients with less severe CAD. However, our study has limitations, including its retrospective design, single-center setting, and small sample size. Further studies in larger and more diverse patient populations are needed to confirm and generalize our results.
